# A Decision Rule for Determining the Optimal Transplant Listing Window for Patients With a Fontan Physiology

**DOI:** 10.1177/23814683211057472

**Published:** 2021-11-15

**Authors:** Laura Delaney

**Affiliations:** Kings Business School, Kings College London, London, UK

**Keywords:** Fontan physiology, heart transplantation, optimal timing

## Abstract

The Fontan is a complex surgical procedure used as a palliative treatment for children with univentricular hearts. In the past, the mortality rate was high and the associated comorbidities as a result of the Fontan circulation were many. However, as research into the condition developed, better understanding has led to a massive reduction in early mortality and a rapidly increasing population of such patients surviving well into adulthood. This has led to a large surge in patients with congenital heart disease being referred for cardiac transplant assessment. According to research, listing these patients at the optimal time is the key to improving transplant outcomes. However, determining that optimal time is unclear and controversial. In this article, I address this issue by developing an optimal timing rule that accounts for the factors faced by specialist cardiologists in determining when transplant ought to be considered for this cohort of patients.

## Introduction

The Fontan is a surgical procedure that was initially developed in 1971 as a palliative treatment for children born with a heart with only one ventricle.^1^ The technicalities of the procedure are complex, and it was initially associated with very high mortality rates and significant postoperative complications. However, as research into the procedure, and the associated condition of the patient with acquired Fontan circulation progressed, mortality rates have declined, and in the majority of cases, patients grow and develop in a near-normal way and live with a good quality of life.^2^ Coats and colleagues^3^ have reported a “5-fold increase in Fontan patients, with a projected 60% increase over the next decade,” while Khairy and colleagues^4^ and Gamba and colleagues^5^ report that Fontan surgery survival exceeds 80% at 20 years postsurgery. However, they also point out that eventual failure of the Fontan physiology is inevitable prompting referral of the patient for assessment for heart transplantation. Indeed, over the past 15 years, there has been a 40% increase in transplantation of adults who were born with a heart condition (Congenital Heart Disease [CHD]),^*^ and while the current proportion of transplants performed for Fontan failure is unclear, it is predicted that these patients will account for 70% to 80% of CHD transplantation in the future.^2,6^

However, according to Crossland and colleagues,^7^“The Fontan group has significant comorbidity with limited options for medical therapy^8–11^ and optimal timing for listing and transplanting these patients is therefore key to improving outcomes.” The point about optimal timing is also mentioned in Kenny et al.,^2^ who say that “determining the optimal timing for transplant in these patients remains unclear” and suggest that having an understanding of this “can help guide decision making in regards to listing.” Currently, the decision about the optimal time to list is based on the specialist clinicians’ experienced judgement, but there is no objective benchmark guiding this decision. “The timing of transplant in the late failing Fontan remains highly controversial and an area of developing expertise.”^2^ Indeed, according to Polyviou and colleagues,^12^ there is a need for tools that help guide them in the difficult decision-making around listing this high-risk group for transplantation.

In this article, I develop a decision rule that clinicians can use to determine the optimal time to list individual patients with a Fontan physiology for heart transplantation. Clinical judgement based on experience is extremely important in the decision to list and should not be discounted, but having such a rule will be an additional tool that can assist them in devising the optimal management strategy for patients with a failing Fontan. The main benefits of the rule I derive are that it is easy to apply and serves as an objective benchmark. The reason this research is so worthwhile is that in those patients with Fontan failure who do survive a transplant, long-term survival is comparable with other diagnoses,^7^ and according to Burch and colleagues,^13^ long-term “cardiac transplant (survival) for adult CHD is better than cardiac transplant for all other causes.” However, to get to this stage, Fontan patients must be listed and subsequently transplanted at the right time.

This rule must account for a number of factors. On the one hand, transplantation carries huge risk for a Fontan patient owing to multiple prior surgeries, immunological sensitivities, and multi-organ involvement.^2^ According to Karamlou and colleagues,^14^“Patients with CHD wait longer on the list than non-CHD patients and carry a higher waiting list mortality.” These considerations, combined with the unrelenting progression of physiological failure should prompt early consideration of transplantation in apparently stable patients^2,15^ before they become too unwell and their risk of death posttransplant (if they get that far) is too high.

On the other hand, listing patients too early is not ideal. Even for apparently stable patients, the surgery is high risk. Posttransplant, the patient is on immunosuppression therapy for the rest of their lives, which places them at high risk of serious infection. Indeed, this is only one of a number of such considerations outlined in Kenny et al.^2^

In terms of the model, the doctor has the choice to list the patient at time *t* or to wait until some future time. In making this decision, three features are very important: 1) *irreversibility* in the sense that once the patient is transplanted, this cannot be reversed^*^; 2) *flexibility*—if the doctor decides not to list the patient at time *t*, she may do so in the future so that it is not a now or never decision; and 3) *uncertainty*—the outcome from performing the transplant is always uncertain ex ante. Thus, the doctor’s choice about whether or not to list a patient is a *real option*; that is, she has the option to list the patient now or she can “watchfully wait,” which preserves the option to list in the future. The important point about real options is that waiting has value owing to the uncertainty of the outcome and the irreversible nature of the decision. The idea is that deferring a decision may allow for more information to be revealed that may be valuable for the decision to list; for example, it may prevent a patient from being put through a very risky procedure in a suboptimal clinical state; or equivalently, the acquisition of more information may justify the decision to transplant in the future. “With deferred treatment, the passing of time offers a medical history and therefore increases information upon which a clinical decision can be made.”^16^ In essence, the intention is to make a confident decision based on as much information as possible and the option to defer is valuable because it allows doctors to observe the progression on the Fontan failure as well as to gain more information about the patient’s clinical status. However, there is a cost to deciding to defer if the progression of the failing state is rapid and the optimal listing window is missed. The clinical status of the patient may deteriorate during the waiting period which may adversely affect the expected outcome. The value of waiting must be derived, via a method known as dynamic programming, by comparing these expected costs against the expected benefit from deferring.

According to Driﬃeld and Smith,^16^ immediate treatment versus watchful waiting has been assessed in other contexts. They give some examples of glue ear, small abdominal aortic aneurysms, among others. However, they also point out that these studies do not model deferral properly because they fail to incorporate the fact that should the clinical status of the patient begin to deteriorate significantly, the option to list can be exercised at any time and patient can be immediately listed. The model in this article overcomes this limitation because it accounts for flexibility over timing in the sense that there are no stipulations over when the patient can be listed. One example of such a stipulation could be that listing can only happen immediately or not at all.

The model is one of sequential sampling because it allows for a multiperiod perspective and will enable me to incorporate all of the dimensions just discussed. A similar approach has been applied previously in the context of health technology adoption.^17^ The approach has also been applied in a wide range of other contexts to problems in, for example, corporate finance,^18^ market microstructure,^19^ migration,^20^ and the environment.^21^ The idea is that the cardiologist has a checklist of symptoms (which she determines) against which the clinical status of an individual patient is checked. These, along with the quality of the symptoms, that is, how good they are at predicting posttransplant outcome, enable the clinician to determine a patient-specific level that can be measured against two threshold values that are analytically derived in this article. If the patient’s level is above the upper threshold, the patient is deemed too well for transplant and will be reassessed in due course. If the level is below the lower threshold, the patient will be considered too unwell for transplant. However, if the level is between the two thresholds, the patient should be listed. During the listing period, while the patient waits for transplant, the patient will be routinely reviewed and his level will be redetermined accordingly. If the patient’s clinical status declines to the point of his level dropping below the lower threshold, he should be removed from the list.

The model accounts for the *rate* of clinical decline, that is, the number of comorbidities developed over a predetermined period. If, for example, that period is 6 months, then a patient who is reviewed after 1 year and has developed 2 comorbidities in that year has rate of decline of 1 comorbidity per 6-month interval. In other words, depending on the length of time between reviews, the number of comorbidities developed will be adjusted to a number per predetermined level; that is, it will be adjusted to a rate. This ensures consistency across patients who are reviewed at different intervals.

In principle, if their health subsequently improves, the patient will be reviewed and the decision rule reapplied. If the clinical status is such that he is well enough to be listed again, he can be relisted. However, owing to the nature of the disease progression of a failing Fontan, this is unlikely to happen in practice very often. Moreover, it is worth pointing out that, for cardiac transplantation in the United Kingdom, at least, it is not the practice for a patient to be listed if the clinician knows that if the patient were to be offered an organ, they would not be able to accept it. Indeed, if a listed patient develops an infection, they will be removed from the list, even for just a few days, and relisted once the infection clears.

The policy structure just described represents what happens in practical settings. Specialist cardiologists list patients they deem to be ill enough, but not too ill. As discussed above, if a listed patient becomes too ill, they are removed from the list. The analytic aim of this article is to determine points to delineate the boundaries of these regions.

I show that the longer the patient is expected to wait on the list before getting an organ, the shorter is his life expectancy if he does not have the transplant, and the faster his rate of clinical decline, the earlier he should be listed, that is, while he is still reasonably well. These results are plausible and intuitive and suggestive of the model’s usefulness in practice.

This article represents the first stage of a broader research agenda to determine a decision criteria with regard to optimal timing that specialist cardiologists can use in clinical practice. I demonstrate in this article the theoretical model underpinning the rule and examine it hypothetically. However, to make it fit for practice, the model needs to be incorporated in a simple software program that calculates the optimal decision once clinicians input the various parameter values pertaining to a specific patient. Moreover, some input parameters will require empirical estimation from past data, which is discussed in a later section and, finally, there are some limitations to the current model that are discussed in the Conclusion. The model should be adapted so that there are future versions of it that account for these limitations and the software program updated accordingly.

I also wish to point out that the practical tool is intended for use by cardiologists at specialist centers in which there is the expertise and risk appetite for transplanting patients with Fontan physiology. While there are a number of centers in the United Kingdom, for example, that perform cardiac transplants, only the Freeman Hospital in Newcastle upon Tyne performs transplants on patients with Fontan physiology. As such, it is the clinicians at that center that make the decision about listing such patients. Finally, it is worth pointing out that the model here is derived with the listing of Fontan patients for heart transplantation in mind. However, the techniques used are not specific to this issue and could be applied, in principle, to any decision about when to treat a patient in a vast array of clinical settings.

The rest of the article is organized as follows. In the second section, the description of the model is outlined. In the third section, the decision criteria are derived and are explained via the use of an example in the fourth section. In the fifth section, I discuss the model in terms of its plausibility for clinical use and also how the input parameters required can be estimated empirically. The sixth section concludes with a discussion of some of some of the limitations of the current model that ought to be incorporated into future versions of the model.

## The Model Outline

At some time *t*, the doctor considers whether or not to list a patient for cardiac transplant. Note that *t* represents the time of any assessment that may come after or include the initial assessment, where t=0 is the initial assessment. If the patient is listed and subsequently, transplanted, his outcome will be Good or Bad. If the outcome is Good, the patient has long-term transplant survival leading to a life expectancy of $L_{T}>0$ . In this case, his life expectancy from having the transplant significantly exceeds his life expectancy from not having it. I denote the life expectancy from not having the transplant by $L_{\mathrm{NT}}$ , such that $0<L_{\mathrm{NT}}<<L_{T}$ . If, however, the outcome is Bad, he dies during or shortly after the surgery and his long-term life expectancy is zero.

Prior to transplant and, therefore, prior to listing, there is uncertainty over the outcome of the transplant for that patient. At the time of the first assessment, before any tests on the patient have been conducted, the doctor has a prior belief of $p_{0}=50\%$ that the patient’s outcome will be Good.

Tests are conducted and the results of the tests serve as *signals* that are indicative of the patient’s posttransplant outcome and, thus, help alleviate some of the doctor’s uncertainty. There is a standard checklist for all patients undergoing such assessment against which their comorbidities are checked. This checklist accounts for the factors suggested by Kenny and colleagues,^2^ which must be considered (e.g., multi-organ involvement, immunological sensitivities, and number of prior surgeries). Hereafter, I use the terms signals, comorbidities, and symptoms synonymously. For example, Patient *i* has the symptoms checked Yes in Table 1, and the existence (or not) of each symptom is deemed to be indicative of a Good or Bad outcome.

**Table 1:** Example of Symptom Checklist

Symptom	Yes/No	Good/Bad
Protein losing enteropathy	Yes	Bad
Preserved ventricular function	Yes	Bad
Exercise tolerance ≥ Level X	Yes	Good
Central venous pressure ≥ Level Y	No	Good
Number of prior surgeries ≥Z	Yes	Bad
Severe liver cirrhosis	No	Good
Kidney function: Creatinine ≥ Level A	Yes	Bad
Healthy body mass index	Yes	Good
Support network	Yes	Good
High antibodies (= long wait expected)	Yes	Bad

In this example, the number of signals indicative of a Good outcome (i.e., five symptoms) is exactly equal to the number of signals indicative of a Bad outcome. However, it is the *quality* of the signals that matter. Signals are deemed to be of high quality if they are a correct reflection of the true outcome. For example, if the acquisition of protein losing enteropathy (PLE) is indicative of a Bad posttransplant outcome, and the outcome of a high percentage of patients with PLE turns out to be Bad, then PLE is a high quality signal. In the model, however, the quality parameter, which is denoted by θ , is not signal specific. I discuss in more detail in the discussion section how the overall quality of the signals could be estimated holistically, but for now, the signal quality is interpreted as the percentage of patients for whom the signals are accurate predictors of the posttransplant outcomes. If this proportion is high, the signal quality is high.

Another important point about the comorbidities is that some are, naturally, more important than others. This is easily accounted for by considering a specific comorbidity in terms of its associated comorbidities. Take PLE for example. This comorbidity could be considered in terms of, for example, 1) level of albumin <A , 2) massive ascites, 3) peripheral edema, 4) length of time since diagnosis >L years, 5) effectiveness of other palliative treatments, and so on. If patient A has 1, 2, 3, and 4, these are four bad signals associated with PLE. If, however, patient B has 2 and 4, then this represents two bad signals associated with PLE, so while both have PLE, patient A is sicker with it than patient B. In this way, the importance of specific comorbidities would be relative and, importantly, the model would account for how a specific comorbidity affects one patient relative to another.

For each patient, the number of signals, as well as their quality, will determine, at the time of assessment t , the doctor’s belief (i.e., probability) on a Good outcome posttransplant. This probability is denoted by $p_{t}$ . In the next section, I derive two decision bounds, denoted by $p_{H}$ and $p_{L}$ with $p_{H}>p_{L}$ , against which $p_{t}$ will be measured.

With regard to the decision bounds, let $p_{L}$ denote the lower bound and $p_{H}$ the upper bound. $p_{H}$ is the boundary between the clinical status of a patient that is too well and one who ought to be listed. $p_{L}$ is the boundary between the clinical status of a patient that should be listed and a patient that is too unwell.

Patients can develop a new comorbidity at any time, and if they do, the doctor’s belief in a Good outcome is revised accordingly. For a patient that is listed, if their clinical status declines such that the belief $p_{t}$ crosses the $p_{L}$ threshold, then they are removed from the list. For a patient that was deemed too well to be listed, if they develop more comorbidities, $p_{t}$ may cross the $p_{H}$ threshold so that the patient is listed. However, when a patient is listed, it is because there are no more treatment options available and the patient’s clinical status is deemed not to improve in any meaningful way. As such, it is very unlikely that he will recover enough to be removed from the list by becoming too well. The derivation of $p_{H}$ in the section “Derivation of $p_{H}$ ” is underpinned by this assumption.

The decision bounds will account for a number of other factors. For example, the expected waiting time on the list is one such factor. If the patient is listed at time t , he will receive his transplant at some uncertain future time $t_{T}$ so that the expected length of time on the list is $\delta :=t_{T}-t$ . It is, of course, logical to assume that $L_{\mathrm{NT}}>\delta$ ; in other words, that his expected waiting time on the list does not exceed his life expectancy without the transplant.

The cost, in terms of life expectancy, of being listed is the patient’s current life expectancy without the transplant (defined earlier as $L_{\mathrm{NT}}$ ) less the amount of time on the list, that is, $C=L_{\mathrm{NT}}-\delta$ . For example, say $L_{\mathrm{NT}}=1$ year and the patient spends 8 months on the list so that δ=0.67 years. If the outcome is Bad and the patient dies shortly after the transplant, then the patient loses 4 months of life from being listed; that is, C=1−0.67=0.33 years.

Another such factor is the rate of clinical decline of the patient. The patient’s development of further comorbidities is random. I let the the number of comorbidities they develop in, say, a 1-year horizon be denoted by μ .

An important assumption underpinning the decision criteria is that the doctor making the decision is risk-neutral. The motivation for this is that the decision tool derived in this article provides an objective benchmark for all such specialist clinicians to use which does not take into account individual doctors’ appetite for risk. This ensures that by adhering to the rule, the decision by the doctor to list a patient is based solely on the factual information she has available about the patient’s clinical status and is not influenced by her subjective preferences. Hence, two patients with little clinical difference but with different doctors may receive opposing management strategies if the doctors do not adhere to such an objective criteria because, as long as this is the case, the doctor’s own appetite for risk would be a driving force underpinning the patient’s treatment. The discount rate used by the doctor is denoted by r .

This section outlined in detail the input parameters to the model that the doctors will use in determining the best decision with respect to an individual patient. The technical analysis will follow in the next section. However, for the reader’s convenience, I provide a comprehensive table in the online appendix that summarizes the discussion in this section.

## Derivation of the Decision Criteria

In determining the optimal management strategy, the doctor chooses the strategy that maximizes the life expectancy of the patient. In this section, I derive the decision bounds $p_{H}$ and $p_{L}$ , described in the section “The Model Outline.”

Suppose that at some point in time, the belief in a Good outcome is given by $p_{t}$ . Before proceeding with the derivation of $p_{H}$ and $p_{L}$ , I derive the expression for $p_{t}$ using Bayes’ rule. One thing to note is that the signals (i.e., the comorbidities that are acquired) are binomially distributed random variables with parameters θ and n , where θ has been defined previously and n are the number of signals. Furthermore, I assume that if the patient is too well for transplant in the sense that they have developed few comorbidities (i.e., $k_{t}$ exceeds the upper threshold $k_{H}$ , to be defined later), transplanting them would be a Bad outcome because the risk of the operation is too high relative to their clinical status. Therefore, in this instance, the acquisition of a comorbidity is indicative of a Good posttransplant outcome because the benefit to them of a Good posttransplant outcome is much higher than not having the transplant. Hence, the probability in a Good outcome posttransplant, conditional on the patient having acquired b comorbidities out of a possible n on the checklist is given by

(1) $$\begin{array}{c} p_{t}=\frac{\theta^{b}{\left(1-\theta \right)}^{n-b}p_{0}}{\theta^{b}{\left(1-\theta \right)}^{n-b}p_{0}+{\left(1-\theta \right)}^{b}\theta^{n-b}(1-p_{0})}\\ \;=\frac{{\left(1-\theta \right)}^{k_{t}}}{\theta^{k_{t}}+{\left(1-\theta \right)}^{k_{t}}} \end{array}$$

where $k_{t}:=n-2b=$ Number of Good symptoms in excess of the Number of comorbidities at time t and $p_{0}=1/2$ as mentioned in the previous section.

However, once a patient is listed, it is because they have acquired enough comorbidities relative to the upper threshold. When this is true, the acquisition of any further comorbidities implies they are progressively worsening to the point where they may be too ill. Hence, in the listing region, the acquisition of a comorbidity is indicative of a Bad posttransplant outcome. To account for this, θ and 1−θ are interchanged in the derivation of $p_{t}$ above so that, in the listed region, the probability of a Good posttransplant outcome is $\left(1-p_{t}\right)$ .

I rewrite $p_{t}$ as $p(k_{t})$ to highlight the dependence of $p_{t}$ on $k_{t}$ , where $k_{t}$ is defined above and can be interpreted as the clinical status of the patient at time t . Indeed, we can rewrite Equation (1) so that it is given in terms of $k_{t}$ as follows:

(2) $$k_{t}=\frac{\ln\left(\frac{1-p_{t}}{p_{t}}\right)}{\ln\left(\frac{\theta }{1-\theta }\right)}$$

such that θ∈(0,1) and $p_{t}\in (0,1)$ .

In the section “Derivation of $p_{H}$ ,” I derive the upper decision bound $p_{H}$ , and in the section “Derivation of $p_{L}$ ,” I derive $p_{L}$ . Note that $k_{H}$ and $k_{L}$ are the clinical status bounds associated with $p_{H}$ and $p_{L}$ , respectively, as per Equation (2).

### Derivation of $p_{H}$

To derive $p_{H}$ , we must consider the clinical status of the patient in three different scenarios/regions.

**Region 1:** In this region, the clinical status of the patient is such that they should be listed immediately; that is, $k_{t}\leq k_{H}$ .

His expected total life expectancy (TLE) from being listed now with an expected waiting time of δ is given by

(3) $$\begin{array}{c} V_{R1}(k_{t})=\left(1-p(k_{t})\right)(L_{T}-L_{\mathrm{NT}}+\delta )+p(k_{t})\left(0-(L_{\mathrm{NT}}-\delta )\right)\\ =\left(1-p(k_{t})\right)L_{T}+\left(\delta -L_{\mathrm{NT}}\right) \end{array}$$

where $\delta <L_{\mathrm{NT}}$ . If it were not the case that $\delta <L_{\mathrm{NT}}$ , then the patient’s life expectancy without the transplant is less than his expected waiting time, and as such, listing would have no value as it would be unlikely they would live long enough to get the transplant.

**Region 2:** If this region, the clinical status of the patient is such that they are much too well for transplant at the time of assessment; that is, $k_{t}>k_{H}+1$ , where $k_{H}$ denotes the net number of symptoms yielding the belief level $p_{H}$ . They have the option of being listed in the future if their clinical status declines significantly. The value of such option has been derived in previous studies,^18,22^ but with two main differences: 1) if the patient’s clinical status improves even more because, for example, after the assessment, they are offered a new treatment which cures any comorbidity they may have had prior to assessment, the option to list them is less valuable; and 2) a Bad posttransplant outcome is associated with few comorbidities for the reason explained above.

It is given by

(4) $$V_{R2}(k_{t})=\frac{\mu }{r+\mu }\left[\left(2p(k_{t})\theta +1-\theta -p(k_{t})\right)V_{R2}(k_{t}-1)+\left(p(k_{t})+\theta -2\theta p(k_{t})\right)V_{R2}(k_{t}+1)\right]$$

subject to the condition that as $k_{t}\to \infty$ , $V_{R2}(k_{t})\to 0$ . A general solution, subject to this condition, is given by

(5) $$V_{R2}(k_{t})=\frac{A\beta_{1}^{k_{t}}}{\theta^{k_{t}}+{\left(1-\theta \right)}^{k_{t}}}$$

where A>0 is some constant to be determined and $\beta_{1}$ is the smaller root of the quadratic equation

(6) $$\beta^{2}-\left(\frac{r+\mu }{\mu }\right)\beta +\theta (1-\theta )=0$$

and $0<\beta_{1}<\theta$ .

**Region 3:** In this region, the patient is too well for transplant, but if they acquire one more comorbidity, they should be listed; that is, $k_{H}<k_{t}\leq k_{H}+1$ . The value of the option is given by Equation (4), but with $V_{R2}(k_{t}-1)$ replaced with $V_{R1}(k_{t}-1)$ .

(7) $$\begin{array}{c} V_{R3}(k_{t})=\frac{\mu }{r+\mu }\left[\left(2p(k_{t})\theta +1-\theta -p(k_{t})\right)V_{R1}(k_{t}-1)+\left(p(k_{t})+\theta -2\theta p(k_{t})\right)V_{R2}(k_{t}+1)\right]\\ \;=\frac{\mu }{r+\mu }\left[\left(\frac{(1-\theta )\theta^{k}+\theta {\left(1-\theta \right)}^{k}}{\theta^{k_{t}}+{\left(1-\theta \right)}^{k_{t}}}\right)\left(\left(1-p(k_{t}-1)\right)L_{T}+\delta -L_{\mathrm{NT}}\right)+\frac{A\beta_{1}^{k_{t}+1}}{\theta^{k_{t}}+{\left(1-\theta \right)}^{k_{t}}}\right] \end{array}$$

Note that this corresponds with Equation (11) from Thijssen and colleagues,^22^ but with their $k_{t}+1$ replaced with $k_{t}-1$ and vice versa. The reason is that in their model, stopping is optimal (i.e., investing) after one more good signal, whereas in my model, the stopping region will be entered (i.e., it becomes optimal to list) after one more bad signal (i.e., the acquisition of one more comorbidity).

To determine $p_{H}$ , we solve for the following conditions (cf. Thijssen et al.,^22^ p. 7):

(8) $$V_{R1}(k_{H})=V_{R3}(k_{H})$$

and

(9) $$V_{R2}(k_{H}+1)=V_{R3}(k_{H}+1)$$

which yield

(10) $$\begin{array}{c} p_{H}={\left[\Psi \left(\frac{L_{T}}{L_{\mathrm{NT}}-\delta }-1\right)+1\right]}^{-1} \end{array}$$

where

(11) $$\Psi :=\frac{\left(r+\mu (1-\beta_{1})\right)\left(r+\mu \theta \right)-\mu^{2}\theta (1-\theta )}{\left(r+\mu (1-\beta_{1})\right)\left(r+\mu (1-\theta )\right)-\mu^{2}\theta (1-\theta )}$$

This belief is well-defined for $0<p_{H}\leq 1$ . It is straightforward to verify that it is well defined for Ψ≥0 since $\delta <L_{\mathrm{NT}}$ and $L_{T}>>L_{\mathrm{NT}}$ , as previously discussed. I show in the online appendix that Ψ≥0 . Thus, $p_{H}$ is well-defined.

### Derivation of $p_{L}$

To derive $p_{L}$ , we must consider three additional regions to those described above.

**Region 4:** Say the clinical status of the patient is such that $k_{L}+1<k_{t}\leq k_{H}$ . This implies that he should be listed immediately, and even if he acquires one more comorbidity, he will still not be removed from the list. The value to the patient in this state is given by the expected life expectancy from getting the transplant δ periods in the future plus the value of option to be removed from the list should his status worsen significantly.^*^

The value of the option to remove the patient from the list is derived in a similar way to that in Region 2. However, I assume that only negative signals arrive in this region; that is, once $k_{t}$ reaches $k_{H}$ from above. In other words, it is very implausible to assume that the patient will recover enough to be removed from the list by being deemed too well once he is listed. Recall, moreover, that since the patient is listed in this region, we replace $p(k_{t})$ with $1-p(k_{t})$ in Equation (4) ( $p(k_{t})$ defined by Equation 1).

This gives

(12) $$\begin{array}{c} V_{O4}(k_{t})=\frac{\mu }{r+\mu }\left(p(k_{t})+\theta -2\theta p(k_{t})\right)V_{O4}(k_{t}-1) \end{array}$$

subject to the condition that $\underset{k_{t}\to -\infty }{\lim}V_{O4}(k_{t})=\infty$ . Intuitively, the more comorbidities that are acquired while waiting, the more valuable is the option to delist.

A general solution, subject to this condition is given by

(13) $$V_{O4}(k_{t})=\frac{B\beta_{2}^{k_{t}}}{\theta^{k_{t}}+{\left(1-\theta \right)}^{k_{t}}}$$

where B is some constant to be determined and $\beta_{2}$ solves

(14) $$-\left(\frac{r+\mu }{\mu }\right)\beta_{2}+\theta (1-\theta )=0$$

(cf. Equatios 7 and 8 in Thijssen et al.,^22^ but recall that we do not consider positive signals so that their F(k+1) is zero in the context of this case.)

It is easily verified from Equation (14) that $\beta_{2}<\theta$ and $\beta_{2}<(1-\theta )$ . Therefore, by Equation (13), the boundary condition is satisfied; that is, $\underset{k_{t}\to -\infty }{\lim}V_{O4}(k_{t})=\infty$ .

Therefore, the total value of being listed in Region 4 is given by the combined value from his expected life expectancy from being listed plus the value of the option to delist. Note that the expected life expectancy from being listed is given by Equation (3).

(15) $$V_{R4}(k_{t})=(1-p(k_{t}))L_{T}+\left(\delta -L_{\mathrm{NT}}\right)+\frac{B\beta_{2}^{k_{t}}}{\theta^{k_{t}}+{\left(1-\theta \right)}^{k_{t}}}$$

**Region 5:** Say the clinical state of the patient is such that he is currently listed, but if he acquired one more comorbidity, he should be removed from the list; that is, $k_{L}<k_{t}\leq k_{L}+1$ . If he is removed from the list, his life expectancy is $L_{\mathrm{NT}}$ . Therefore, the option to de-list must satisfy Equation (12) with $V_{O4}(k_{t})$ replaced by $V_{O5}(k_{t})$ and $V_{O4}(k_{t}-1)=L_{\mathrm{NT}}$ . Therefore,

(16) $$\begin{array}{c} V_{O5}(k_{t})=\frac{\mu }{r+\mu }\left(p(k_{t})+\theta -2\theta p(k_{t})\right)L_{\mathrm{NT}} \end{array}$$

and the total Region 5 value is given by

(17) $$V_{R5}(k_{t})=(1-p(k_{t}))L_{T}+\left(\delta -L_{\mathrm{NT}}\right)+\frac{\mu }{r+\mu }\left(p(k_{t})+\theta -2\theta p(k_{t})\right)L_{\mathrm{NT}}$$

This is underpinned by the same intuition as that for Equation (15). However, the difference is that in Region 4, the acquisition of one more comorbidity does not result in removal from the list, whereas it does in Region 5. As such, the value of the options to delist differs in these regions.

**Region 6:** Say $k_{t}\leq k_{L}$ . In this case, the patient is deemed too unwell for transplant so he will not be offered a place on the list. The value is represented by his life expectancy without the transplant

(18) $$V_{R6}(k_{t})=L_{\mathrm{NT}}$$

To determine $p_{L}$ , the following boundary conditions must be satisfied:

(19) $$V_{R6}(k_{L})=V_{R5}(k_{L})$$

and

(20) $$V_{R5}(k_{L}+1)=V_{R4}(k_{L}+1)$$

Replacing for $V_{R4}$ , $V_{R5}$ and $V_{R6}$ in these boundary equations (using Equations 15, 17, and 18, respectively) gives, after some algebraic manipulation,

(21) $$p_{L}=\frac{(r+\mu )\left(\delta +L_{T}\right)-\left(2r+\mu (2+\theta )\right)L_{\mathrm{NT}}}{(r+\mu )L_{T}+\mu \left(1-2\theta \right)L_{\mathrm{NT}}}$$

This threshold is a probability level. Thus, to be well-defined, it must be that $0<p_{L}\leq 1$ . It is justifiable to assume that the long-term life expectancy posttransplant significantly exceeds the life expectancy without the transplant; that is, $L_{T}>>L_{\mathrm{NT}}$ in order for the risk of the operation and posttransplant complications to be worthwhile. Moreover, I assumed previously that $\delta <L_{\mathrm{NT}}$ . Given this, it is easy to verify that $p_{L}$ is well-defined.

## Listing Criterion

The listing criterion can be given in terms of $p_{t}$ or $k_{t}$ . However, since $k_{t}$ is easily observable by the doctor as the number of Good signals in excess of Bad signals, we give the criterion in terms of $k_{t}$ . However, as explained in the previous section, $p(k_{t})$ as given by Equation (1), must be replaced by $1-p(k_{t})$ for the analysis of the lower threshold. Therefore,

(22) $$k_{L}=\frac{\ln\left(\frac{p_{L}}{1-p_{L}}\right)}{\ln\left(\frac{\theta }{1-\theta }\right)}$$

where $p_{L}$ is given by Equation (21). Moreover, from Equation (2),

$$k_{H}=\frac{\ln\left(\frac{1-p_{H}}{p_{H}}\right)}{\ln\left(\frac{\theta }{1-\theta }\right)}$$

and

$$p_{H}={\left[\Psi \left(\frac{L_{T}}{L_{\mathrm{NT}}-\delta }-1\right)+1\right]}^{-1}$$

with Ψ given by Equation (11).

The optimal timing decision rule is given by

(23) $$\mathrm{Criterion}=\;\left\{ \begin{array}{cc} \mathrm{if}\;k_{t}\geq \lceil k_{H}\rceil & \mathrm{Too}\;\mathrm{Well}\\ \mathrm{if}\;\lceil k_{L}\rceil \leq k_{t}\leq \lfloor k_{H}\rfloor & \mathrm{List}\\ \mathrm{if}\;k_{t}\leq \lfloor k_{L}\rfloor & \mathrm{Too}\;\mathrm{Unwell} \end{array}\right.$$

Note that working out $k_{H}$ and $k_{L}$ will yield values that can be any real number, when they should be discrete. As such, the criterion is stated in terms of ceiling and floor functions where, for some x∈R , ceil(x)=⌈x⌉:=min{y∈N|y≥x} ; that is, as the smallest integer greater than x , and floor(x)=⌊x⌋:=max{y∈N|y≤x} ; that is, the largest integer less than x .

**Example 1:** Consider patient X who presents with the following characteristics. They are developing an average of μ=2 comorbidities per year, the quality of their symptoms as being a correct reflection of their posttransplant outcome is θ=80% , r=10% , the patient’s expected waiting time while on the list is δ=0.5 year, their posttransplant long-term life expectancy is $L_{T}=10$ years, and if they do not get the transplant, they are expected to live for $L_{\mathrm{NT}}=2$ years. Plugging this information into Equations (10) and (21) and from there into Equations (2) and (22), respectively, gives $k_{H}=2.93$ and $k_{L}=0.18$ . Thus, the patient should be listed if $k_{t}\leq \lfloor k_{H}\rfloor =2$ , but if $k_{t}\geq \lceil k_{H}\rceil =3$ he is too well for now. If, however, $k_{t}\leq \lfloor k_{L}\rfloor =0$ , he is too sick to be offered a place on the list, or if listed, he should be removed from the list.

## Discussion

In this section, I demonstrate that the implications obtained from the theoretical model indicate its plausibility for clinical use, and second, I discuss how the input parameters that are required could be estimated.

**Figure 1:**
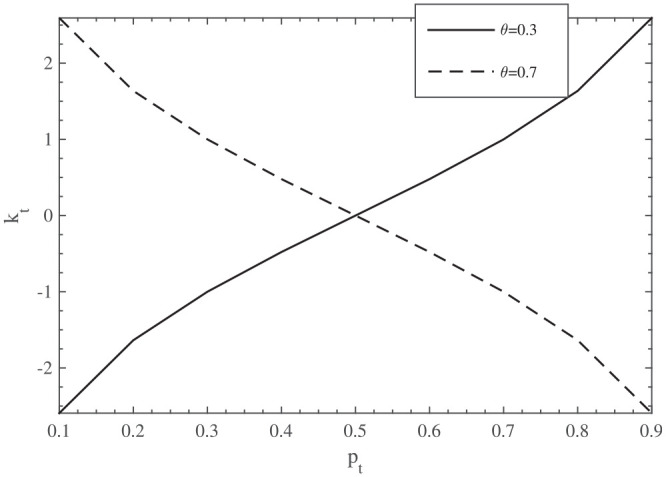
The effect of $p_{t}$ on $k_{t}$ .

First, however, I make the following important point about the quality parameter θ . $k_{t}$ increases in $p_{t}$ for θ<1/2 and decreases in $p_{t}$ for θ>1/2 (see Figure 1). For θ=1/2 , $k_{t}$ is undefined.

As such, the model’s predicted effects of the parameters $L_{\mathrm{NT}}$ , δ , and μ depend on whether we assume θ>1/2 or θ<1/2 .

The Scientific Registry of Transplant Recipients (SRTR) maintains 43 risk-adjustment models for assessing transplant program performance in the United States.^23^ A feature of these models is the importance of certain comorbidities in predicting transplant outcome (analogous to the signal quality in this model). Snyder and colleagues^23^ assess the performance of the SRTR models and find that, in general, posttransplant outcomes are difficult to predict. The C-statistics determining the models’ ability to distinguish between high- and low-risk transplants are wide-ranging and can be low. For the heart models, the C-statistics range between 0.67 and 0.83. Nevertheless, it is reasonable to assume that if the clinician has, based on their own experience and from the literature, chosen to include certain comorbidities in the checklist, they must be good predictors of the posttransplant outcome. Hence, I think it is appropriate to interpret the effects of the above-mentioned parameters for θ>1/2 .

### Model Implications

**Figure 2:**
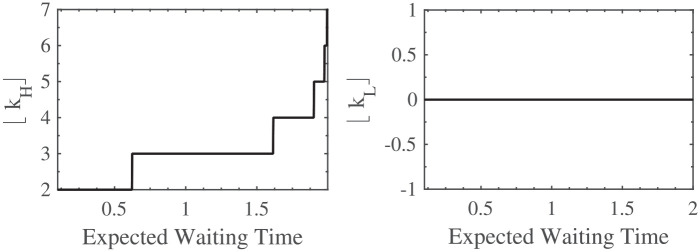
The effect of δ on $\left\lfloor k_{H}\right\rfloor$ and $\left\lfloor k_{L}\right\rfloor$ .

**Figure 3:**
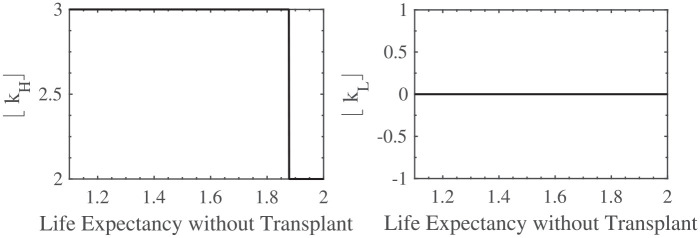
The effect of $L_{\mathrm{NT}}$ on $\left\lfloor k_{H}\right\rfloor$ and $\left\lfloor k_{L}\right\rfloor$ .

**Figure 4:**
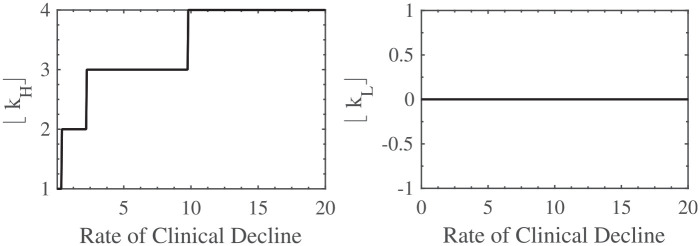
The effect of μ on $\left\lfloor k_{H}\right\rfloor$ and $\left\lfloor k_{L}\right\rfloor$ .

In Figures 2, 3, and 4, I show the impact, according to the model, of the expected waiting time, the patient’s life expectancy if they do not have the transplant, and their rate of clinical decline, respectively, on the optimal listing strategy. To interpret the findings, note that the higher the value of $\left\lfloor k_{H}\right\rfloor$ , the earlier it is optimal to list the patient, and the higher the value of $\left\lfloor k_{L}\right\rfloor$ , the earlier it is optimal to remove the patient from the list. Earlier listing and delisting correspond with a better clinical status. Furthermore, the chosen parameter values are those given in Example 1.

From Figure 2 (left hand plot), the model suggests that the longer the patient is expected to wait on the list before an organ becomes available, the earlier he should be listed; that is, while he is still relatively well. This makes sense because if the doctor were to wait too long, she risks listing the patient when his clinical status is poor, and in that case, with a long expected waiting time, the patient may not survive long enough to get the organ.

On the other hand, from the right hand plot of Figure 2, if the patient is listed, a change in the expected waiting time has no impact on the delisting threshold. Hence, if their comorbidities are not too many, they should remain on the list, irrespective of the expected waiting time. ( δ actually has a positive impact on $k_{L}$ , but its effect is too small to impact $\left\lfloor k_{L}\right\rfloor$ .)

From Figure 3, the shorter is the patient’s life expectancy if he does not have the transplant, the sooner he should be listed. This is again, intuitive, because of the expected waiting time. Another way of looking at this is that if the patient’s life expectancy is relatively long without the transplant, he has time to wait, and therefore, he does not have to be listed immediately.

Furthermore, a change in $L_{\mathrm{NT}}$ does not affect $\left\lfloor k_{L}\right\rfloor$ , and according to the model, the patient should remain listed as long as the comorbidities are not too many.

Finally, from Figure 4, the more comorbidities the patient is acquiring per year, the earlier he should be listed; that is, if his clinical status is quickly deteriorating, he should be listed promptly. Once again, this implication suggested by the model is plausible and intuitive. Moreover, it upholds the suggestion by Kenny et al.^2^ and Everitt et al.^15^ that the unrelenting progression of physiological failure should prompt early consideration of transplantation in apparently stable patients.

However, a change in the rate of clinical decline has no impact on the threshold at which the patient should be removed from the list. This implies that if the patient is listed, he should remain so until enough comorbidities are realized (i.e., until $k_{t}\leq \lfloor k_{L}\rfloor$ ), even if his clinical status is declining quickly.

To summarize, the three important factors that the doctor ought to consider when deciding the best management strategy for a given patient are the rate of clinical decline, the patient’s life expectancy if he does not have the transplant, and the length of time he is expected to wait on the list are all incorporated into the model. The effects of these factors on the optimal listing decision implied by the model are plausible and realistic, which further implies that this model is a credible one for use in clinical practice.

### Parameter Estimates

To calculate the $k_{H}$ and $k_{L}$ for each patient, we need to estimate $L_{T}$ and $L_{\mathrm{NT}}$ . Note that in the model, $L_{T}$ and $L_{\mathrm{NT}}$ are referred to in terms of survival, but in practice, $L_{T}$ and $L_{\mathrm{NT}}$ could alternatively be measured in terms of QALYs (quality-adjusted life years), which is the standard measure used in economic evaluation of health care to assess the value of medical interventions. Whatever measure is used, the data for these values are available from the transplant unit.

We also need to estimate δ and μ . μ is patient specific. It represents the patient’s rate of clinical decline and is the simply the number of comorbidities the patient developed over the past, say, 1year. This is easily determined from the patient’s history.

At any given point in time, the transplant coordinators at the transplant center have a good idea of the average waiting time for a patient about to be placed on the list. Part of their job is to examine the national transplant list on a daily basis so they always have a good understanding of its current status. Furthermore, they can personalize this estimate somewhat depending on the individual patient. For example, they may be able to say that an individual patient placed on the list on a given day is expected to wait on average 2 months. However, this estimate may be different for another patient with unusually high antibodies if they were to be listed on that same day. This is deemed to be a comorbidity because such a patient is much harder to match, and anecdotal evidence suggests they wait much longer than patients with few antibodies. In that case, if such a patient was to be listed immediately, the estimated waiting time for them may be, for example, 2 months, rather than 2 months. Similarly, patients with more comorbidities will be placed on the urgent list rather than the routine list giving them priority. Hence, a patient on the routine list has a longer expected waiting time than a patient on the urgent list. As such, it is up to the transplant coordinators to provide this patient specific estimate for δ in the decision model when being applied to an individual patient.

Finally, we need an estimate for the signal quality parameter θ . This represents the adequacy of the existence (or not) of certain comorbidities as accurate predictors of the posttransplant outcome. It is less straightforward to estimate than the other parameters. Recall, first, that the parameter θ is holistic. Second, there is uncertainty over the quality in the sense that any signal can give a false positive (negative) result. In other words, a signal could be obtained that is indicative of a Good (Bad) outcome, but the true outcome turns out to be Bad (Good). A possible approach is to determine, with the use of past data (which the transplant center can access), the quality of each comorbidity (i.e., signal) from the doctor’s checklist (such as that described by Table 1) as an indicator of outcome. If the sample size is limited, we could use Monte Carlo simulation to obtain further estimates of comorbidities and outcomes. Indeed, Wong and Koff^24^ considered the cost-effectiveness of waiting versus immediate treatment for mild chronic hepatitis C. They used data from a trial and performed some Monte Carlo simulation to estimate prognosis beyond the capacity of the trial. If a signal is deemed to be Bad, for example, then its quality could be estimated as the proportion of patients who had that comorbidity when transplanted and their outcome was indeed Bad. We could then combine these individual qualities into a composite measure of quality that embraces all the relevant comorbidities to the listing decision. This approach is also suggested in a related paper by Driffield and Smith,^16^ who advocate using “Monte Carlo simulation to combine multiple sources of uncertainty into a composite measure of well-being that embraces all considerations relevant to the treatment decision.”

## Conclusion

This article views the decision to list patients with a Fontan physiology for cardiac transplantation as a real option. I develop a model to determine the optimal time to list such patients in response to the various suggestions in the literature that listing these patients at the optimal time is “key to improving outcomes.”^7^ However, as yet, determining the optimal time remains unclear.^2^ This article addresses this need by providing a timing model that can be made easy to use in practice, and is plausible and robust in terms of the underpinning intuition.

However, it is worth pointing out two important limitations to this model that future versions will aim to account for. The first is that there is no distinction between the urgent and routine lists. Patients that are deemed sick enough are placed on the urgent list where they gain priority over less ill patients, even if those patients were listed earlier. A future version of this model could aim to separate the listing region into routine or urgent; that is, whether the patient should be urgently listed or not. This has big consequences in terms of waiting time, but also, patients who are urgently listed must remain in hospital while they wait for the organ, whereas routinely listed patients can wait at home living a relatively more normal life.

A further limitation is the following. The Fontan circulation has a significant impact on the liver owing to increased portal hypertension. In some patients, this can result in severe cirrhosis of the liver, and as such, some patients undergoing transplant after a Fontan are deemed to be in need of a heart and liver transplant. Both organs must be from the same donor and the double transplant is performed as a single operation and the listing must be for two organs; in other words, a patient needing both must be listed as needing both organs. The current model is based on the assumption that the patient in in need of just a heart, but a future version of this model could potentially signal whether the patient should be listed for just a heart or for both organs simultaneously. I would envisage that those patients deemed to be in need of both should be listed earlier (i.e., with fewer overall comorbidities) than those needing just a single organ, according to the decision criteria to be derived.

## Supplemental Material

sj-bib-1-mpp-10.1177_23814683211057472 – Supplemental material for A Decision Rule for Determining the Optimal Transplant Listing Window for Patients With a Fontan Physiology Click here for additional data file. Supplemental material, sj-bib-1-mpp-10.1177_23814683211057472 for A Decision Rule for Determining the Optimal Transplant Listing Window for Patients With a Fontan Physiology by Laura Delaney in MDM Policy & Practice

sj-pdf-2-mpp-10.1177_23814683211057472 – Supplemental material for A Decision Rule for Determining the Optimal Transplant Listing Window for Patients With a Fontan Physiology Click here for additional data file. Supplemental material, sj-pdf-2-mpp-10.1177_23814683211057472 for A Decision Rule for Determining the Optimal Transplant Listing Window for Patients With a Fontan Physiology by Laura Delaney in MDM Policy & Practice

sj-tex-3-mpp-10.1177_23814683211057472 – Supplemental material for A Decision Rule for Determining the Optimal Transplant Listing Window for Patients With a Fontan Physiology Click here for additional data file. Supplemental material, sj-tex-3-mpp-10.1177_23814683211057472 for A Decision Rule for Determining the Optimal Transplant Listing Window for Patients With a Fontan Physiology by Laura Delaney in MDM Policy & Practice

## Notes

* The registry of the the International Society for Heart and Lung Transplantation (ISHLT): Thirty-First Official Adult Heart Transplant Report—2014.

* This does not imply the patient cannot be re-transplanted in the future. While this is rare in cardiac transplantation, it is not impossible. What irreversibility implies in this context is that this specific very high risk transplant operation the clinicians are making a decision about cannot be “undone” once it is performed.

* The patient is better off if the value of this option is low because, in that case, his clinical status is better.
