# Supplementary material for: A Decision Rule for Determining the Optimal Transplant Listing Window for Patients With a Fontan Physiology
Source: MDM Policy Pract. 2021 Nov 15;6(2):23814683211057472. doi: 10.1177/23814683211057472 (PMC8597073; doi:10.1177/23814683211057472)
Supplement: sj-pdf-2-mpp-10.1177_23814683211057472 – Supplemental material for A Decision Rule for Determining the Optimal Transplant Listing Window for Patients With a Fontan Physiology [file sj-pdf-2-mpp-10.1177_23814683211057472.pdf]

A Decision Rule for Determining the Optimal Transplant Listing Window for Patients with a Fontan Physiology - Appendix

Laura Delaney *

Appendix

A Summary Table

*Kings Business School, Kings College London, 30 Aldwych, London WC2B 4BG, UK. Email: laura.delaney@kcl.ac.uk

Table 1: **Summary Table**

**Assumptions**	**Interpretation**
Uncertainty	Outcome of transplant is uncertain at time of listing
Irreversibility	Once transplant is performed, the operation is irreversible if patient rejects the organ
Timing	Patient does not need to wait until next review to be listed: can be listed anytime
Risk neutrality	Deciding doctor is risk neutral

**Parameter**	**Notation**
Long-term post-transplant life expectancy	L_T
Life expectancy without transplant	L_{NT} ($<< L_T$)
Expected waiting time on list	δ ($< L_{NT}$)
Cost of listing (to patient)	$C = L_{NT} - \delta$
Number of signals	n
Number of good and bad signals	g and b, resp.
Quality of signals	θ
Number of comorbidities acquired since last review	μ
Discount rate	r

**Symptom**	**Yes /No**	**Good / Bad**
Protein Losing Enteropathy (PLE)	Yes	Bad
Preserved Ventricular Function (PVF)	Yes	Bad
Exercise Tolerance $\geq$ Level X	Yes	Good
Central Venous Pressure (CVP) $\geq$Level Y	No	Good
Number of Prior Surgeries $\geq Z$	Yes	Bad
Severe liver cirrosis	No	Good
Kidney function: Creatinine $\geq$ Level A	Yes	Bad
Healthy BMI	Yes	Good
Support network	Yes	Good
High antibodies (= long wait expected)	Yes	Bad

B Proof that $\Psi \geq 0$

Let $r = 0$ and $\theta < 1/2$. Then $\beta_1 = \theta$ (because it is the smaller root of Eq. (6), $n(\Psi) = \mu^2(1-\theta)(1-2\theta) > 0$ and $d(\Psi) = 0$, where $n(\Psi)$ and $d(\Psi)$ denote the numerator and denominator of Ψ, respectively.

On the other hand, if $r = 0$ and $\theta > 1/2$ $\beta_1 = 1-\theta$, $n(\Psi) = 0$ and $d(\Psi) = \mu^2\theta(2\theta-1) > 0$.

Let

$$\mathcal{Q}(\beta) := \beta^2 - \left(\frac{r+\mu}{\mu}\right)\beta + \theta(1-\theta) = 0.$$

Hence,

$$\frac{\partial \mathcal{Q}(\beta)}{\partial \beta}\frac{\partial \beta}{\partial r} + \frac{\partial \mathcal{Q}(\beta)}{\partial r} = 0 \Longrightarrow \frac{\partial \beta}{\partial r} = -\frac{\partial \mathcal{Q}(\beta)/\partial r}{\partial \mathcal{Q}(\beta)/\partial \beta}.$$

From this we get that $\partial\beta_1/\partial r < 0$ if $2\beta_1 < (r+\mu)/\mu$. Indeed, since β_1 is the smaller root of Eq. (6), this is true.

This further implies that $\partial n(\Psi)/\partial r > 0$ and $\partial d(\Psi)/\partial r > 0$. Hence, $n(\Psi)$, $d(\Psi)$ and, thus, Ψ will be positive for all $r > 0$ and for all $\theta > 0$.
